# It might not be just an intellectual disability: Change of behavior masking the diagnosis of cancer in a psychiatry unit

**DOI:** 10.1192/j.eurpsy.2021.1032

**Published:** 2021-08-13

**Authors:** O. Nombora

**Affiliations:** Psychiatry And Mental Health Service, Vila Nova de Gaia/Espinho Hospital Centre, Vila Nova de Gaia, Portugal

**Keywords:** intellectualdisability, mentalretardation, Behaviourchanges, cancerdiagnosis

## Abstract

**Introduction:**

Intellectually disabled people are vulnerable to somatic and mental illnesses, often presenting behaviour changes. Moreover, difficulties in describing symptoms can limit their access to healthcare system and adequate treatment.

**Objectives:**

Through a case report, we aim to provide an overview on behaviour changes in people with intellectual disability (ID), emphasizing the screening for organic conditions.

**Methods:**

Description of a clinical case and a qualitative review about the assessment of behaviour changes in persons with ID, using PubMed database.

**Results:**

We present a clinical case of a 57-year-old man with history of ID, alcohol and tobacco abuse and Epilepsy. He had previous acute psychiatric admissions due to behaviour disorganization and irritability. In January he was admitted with disorganized behaviour and caregiver exhaustion, and stabilized with Olanzapine 20mg/day. On the 28th day of hospitalization, he fell of his of bed and suffered a mild traumatic brain injury. Cerebral CT scan revealed two metastatic lesions in the brain. Further investigations found out primary neoplastic lung lesion and multiple metastasis. Afterwards, his relatives mentioned a heavy familiar history of cancer and that he had postural instability signs that they did not value.

**Conclusions:**

Although psychiatric disorders are common in patients with ID, we must always remind that behaviour changes can mask the presentation of an organic disease. Despite a long follow-up in Psychiatry, organic conditions should be considered when patients with ID present behaviour changes. Further studies are needed in the assessment of this particular population to provide proper medical, psychological and social care.

